# Determining Treatment Needs at Different Spatial Scales Using Geostatistical Model-Based Risk Estimates of Schistosomiasis

**DOI:** 10.1371/journal.pntd.0001773

**Published:** 2012-09-13

**Authors:** Nadine Schur, Penelope Vounatsou, Jürg Utzinger

**Affiliations:** 1 Department of Epidemiology and Public Health, Swiss Tropical and Public Health Institute, Basel, Switzerland; 2 University of Basel, Basel, Switzerland; London School of Hygiene & Tropical Medicine, United Kingdom

## Abstract

**Background:**

After many years of neglect, schistosomiasis control is going to scale. The strategy of choice is preventive chemotherapy, that is the repeated large-scale administration of praziquantel (a safe and highly efficacious drug) to at-risk populations. The frequency of praziquantel administration is based on endemicity, which usually is defined by prevalence data summarized at an arbitrarily chosen administrative level.

**Methodology:**

For an ensemble of 29 West and East African countries, we determined the annualized praziquantel treatment needs for the school-aged population, adhering to World Health Organization guidelines. Different administrative levels of prevalence aggregation were considered; country, province, district, and pixel level. Previously published results on spatially explicit schistosomiasis risk in the selected countries were employed to classify each area into distinct endemicity classes that govern the frequency of praziquantel administration.

**Principal Findings:**

Estimates of infection prevalence adjusted for the school-aged population in 2010 revealed that most countries are classified as moderately endemic for schistosomiasis (prevalence 10–50%), while four countries (i.e., Ghana, Liberia, Mozambique, and Sierra Leone) are highly endemic (>50%). Overall, 72.7 million annualized praziquantel treatments (50% confidence interval (CI): 68.8–100.7 million) are required for the school-aged population if country-level schistosomiasis prevalence estimates are considered, and 81.5 million treatments (50% CI: 67.3–107.5 million) if estimation is based on a more refined spatial scale at the provincial level.

**Conclusions/Significance:**

Praziquantel treatment needs may be over- or underestimated depending on the level of spatial aggregation. The distribution of schistosomiasis in Ethiopia, Liberia, Mauritania, Uganda, and Zambia is rather uniform, and hence country-level risk estimates are sufficient to calculate treatment needs. On the other hand, countries like Burkina Faso, Mali, Mozambique, Sudan, and Tanzania show large spatial heterogeneity in schistosomiasis risk, which should be taken into account for calculating treatment requirements.

## Author Summary

More than 200 million people are affected by the snailborne disease schistosomiasis. The main strategy to control schistosomiasis is to regularly treat school-aged children with the drug praziquantel. The frequency of praziquantel treatment depends on the average prevalence of schistosomiasis, which can be defined as low (prevalence <10%), moderate (10–50%), or high (>50%). However, it remains unclear at which geographical scale these prevalence levels should be considered to avoid unnecessary treatments but still comply with local needs. We investigated the effect of the geographical scale for an ensemble of 29 West and East African countries using previously published model-based schistosomiasis risk estimates obtained at high spatial resolution. These estimates allow spatial risk aggregation at different geographical scales (i.e., country, region, district, or pixel level). More than 70 million praziquantel treatments are required every year for school-aged children if countrylevel estimates are used. On a more refined geographical scale (i.e., province), annualized praziquantel treatments increase by 12%. Depending on the averaged schistosomiasis prevalence and the spatial risk variation across a country, the difference in the estimated amount of praziquantel between country-level aggregation and other geographical scales might be very important, as for example in Burkina Faso, Ghana, and Mali.

## Introduction

Schistosomiasis is a snail-borne parasitic disease, which affects more than 200 million people globally based on 2003 population estimates [1]. The main burden is concentrated in sub-Saharan Africa, where schistosomiasis control had been neglected for many decades, and hence country-specific infection rates above 25% are the norm rather than the exception [1]–[3].

The global strategy for the control of schistosomiasis, as recommended by the World Health Organization (WHO), is preventive chemotherapy, that is the repeated, large-scale administration of the antischistosomal drug praziquantel to at-risk populations [4]. Because school-aged children are at highest risk of schistosome infection, preventive chemotherapy is primarily targeted at this population group. The frequency of treatment depends on the level of endemicity, defined as low (prevalence <10%), moderate (10–50%), and high (>50%) based on WHO thresholds [4], [5]. Classification into one of these categories should be done according to schistosomiasis prevalence estimates among the school-aged population. In practice, estimates of schistosomiasis and other helminth infection are often based on old survey data and/or very sparse geographical data. However, historical data are largely obsolete due to ongoing control efforts, ecological transformations, demographic changes, and improved hygiene, among other reasons, while sparse geographical data are not representative for large areas [1], [6]–[9]. In addition, most prevalence estimates lack empirical modeling, which is mandatory to obtain accurate averages of the risk of infection over large geographical regions, taking into account spatial heterogeneity. Even though WHO treatment guidelines are valuable tools to plan and conduct preventive chemotherapy programs, it remains unclear at which geographical scale or other stratifications (e.g., ecological zones) the level of endemicity should be defined to avoid unnecessary treatments, increase cost-effectiveness, and meet local needs.

The aim of this paper was to assess the effect of the geographical scale of schistosomiasis risk estimates on the amount of praziquantel treatment needed in the school-aged population in selected parts of Africa. In contrast to existing treatment need calculations based on crude country prevalence estimates [10], we used spatially explicit, model-based schistosomiasis risk estimates at high spatial resolution [2], [3]. Our modeling framework includes uncertainty measures, and hence allows confidence intervals (CIs) to be determined around predicted treatment needs. These empirical estimates are able to capture potential small transmission hotspots within a country that are typical for schistosomiasis, as the disease is often focally distributed [11], [12], and facilitate spatial aggregation of schistosomiasis risk at different geographical scales. In our analysis, we focus on differences in the total amount of required treatments when risk estimates are known at country, regional, district, or pixel level.

## Methods

### Schistosomiasis Risk Estimates

In our previous work, we determined the spatial distribution of *Schistosoma haematobium* and *S. mansoni* in an ensemble of 29 West and East African countries at a spatial resolution of 5×5 km [2], [3]. We used a geostatistical modeling approach, applied to survey data extracted from the Global Neglected Tropical Disease database (http://www.gntd.org) [13]. Statistical relations of *Schistosoma* prevalence with climatic and other environmental factors were used to obtain model-based predictions at unobserved locations using country-level joint posterior distributions. The median and 50% CIs of the joint posterior distributions were utilized for further analyses.

The risk predictions of Schur and colleagues were originally summarized as country-specific *Schistosoma* infection prevalence estimates adjusted for individuals aged ≤20 years in 2010 [2], [3]. For the current analysis, these data were re-adjusted to the school-aged population (children aged between 5 and 14 years), in line with the WHO definition of the school-aged child [14].

### Population Data

Population count data at 1×1 km spatial resolution in Africa for 2008 were obtained from the LandScan global population database (http://www.ornl.gov/landscan/). Country-specific rates of annual change in population during the period of 2005–2010 were extracted from the United Nations World Population Prospects [15]. Estimates on the percentage of children aged between 5 and 14 years among the total population per country were obtained from the Population Division, provided by the international database of the United States Census Bureau [16]. Population estimates of 2008 were projected to 2010 taking into account estimates of annual population change, readily adjusted to school-aged children. These estimates were then converted to a 5×5 km resolution grid using the geographical information system (GIS) software ArcMap version 9.2 (ESRI) to match the spatial resolution of the geostatistical schistosomiasis risk estimates for West and East Africa.

### Data Aggregation by Administrative Zone

National preventive chemotherapy programs are usually implemented at specific administrative levels, for example at country, province, and district level. Shape files containing geographical information on the administrative boundaries of these levels were downloaded from the Map Library (http://www.maplibrary.org) for our ensemble of 29 West and East African countries. These files were linked in ArcMap with schistosomiasis risk estimates and school-aged population to estimate the number of infected children at pixel-level.

Population-adjusted prevalence at a given administrative region was calculated by summing all infected school-aged children at pixel-level and dividing by the total school-aged population in that region. This calculation takes into account that pixels are not equally populated, and hence do contribute to the area-specific prevalence in the same way.

### Calculation of Praziquantel Treatment Needs

The aggregated and pixel-level data on the school-aged children and population-adjusted prevalence were converted to the required amount of drugs per year (“annualized treatment needs”) using WHO schistosomiasis control guidelines [4], [5]. These guidelines suggest different treatment strategies according to endemicity thresholds. High endemicity is defined by prevalence of at least 50% among school-aged children using parasitological diagnostic tests. Low and moderate endemicity settings are classified by prevalence levels of <10% and 10–50%, respectively.

For high endemicity areas, annual treatment of all school-aged children (and other high-risk groups, e.g., fishermen) is proposed. With regard to treatment needs in the school-aged population, we therefore estimated the amount of annualized praziquantel treatment needs in these areas to be equal to the number of school-aged children. In areas with moderate endemicity, it is recommended to treat school-aged children every other year. Hence, we consider half of the school-aged population for annual praziquantel treatment in our calculation of annualized needs. Areas of low endemicity warrant treatment of school-aged children twice during primary schooling (on entry and just before leaving school). Assuming an average duration of 6 years in primary school, we estimate annualized praziquantel needs by considering one third of the school-aged population for treatment every year.

## Results

### Country-Specific Schistosomiasis Risk and Endemicity Classes

**Figure 1:**
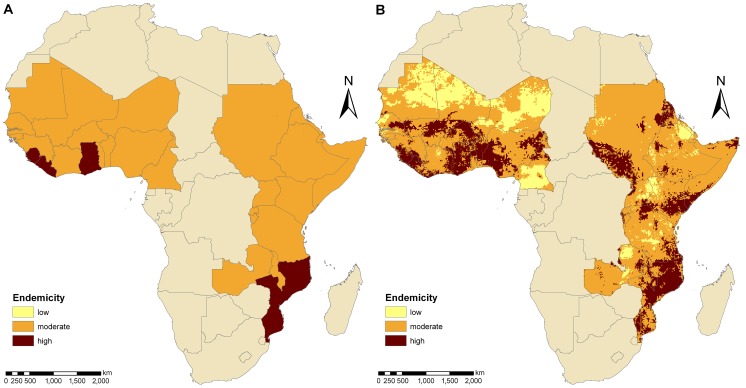
Endemicity estimates of schistosomiasis for an ensemble of 29 West and East African countries. The endemicity levels at country-level (A) and pixel-level (B) are based on previously published geostatistical model-based prevalence estimates [2], [3] and WHO classifications [4], [5]. Low endemicity is defined as schistosomiasis prevalence in school-aged children <10%, moderate endemicity as prevalence between 10% and 50%, and high endemicity as prevalence >50%.

Country-specific estimates on the prevalence of schistosomiasis among the school-aged population and the number of infected school-aged children are summarized in Table 1. The population-adjusted prevalence estimates revealed that 30.2 and 19.5 million school-aged children in West and East Africa, respectively, are infected with *S. haematobium*, *S. mansoni*, or both species concurrently. At the unit of the country, we found infection prevalence ranging between 14.4% (The Gambia) and 60.2% (Liberia) with a mean country-prevalence of 37.3%. Most countries were classified as moderately endemic (prevalence 10–50%), whereas four countries (i.e., Ghana, Liberia, Mozambique, and Sierra Leone) were considered as highly endemic (>50%). Figure 1A displays the schistosomiasis endemicity aggregated at country-level and Figure 1B the spatial distribution of schistosomiasis risk over Africa based on current WHO endemicity thresholds. High- and low-risk areas are found in West and East Africa. Most low-risk areas are located in Cameroon, Saharan Mali, Mauritania, Niger, and parts of Ethiopia, Kenya, Uganda, and Zambia. Large high-risk regions are found along the Niger River, in Ghana, Liberia, Mozambique, and Sierra Leone.

**Table 1:** Country-specific praziquantel treatment needs for the school-aged population estimated at different spatial scales.

Country	Total number of children aged 5–14 years (×10^6^)	Number of *Schistosoma*-infected children aged 5–14 years (×10^6^)	Prevalence adjusted to age group 5–14 years (%)	Treatment needs (10% and 50% cut-offs)				Treatment needs (10% and 25% cut-offs)			
				Country-level (×10^6^)	Province-level (×10^6^)	District-level (×10^6^)	Pixel-level (×10^6^)	Country-level (×10^6^)	Province-level (×10^6^)	District-level (×10^6^)	Pixel-level (×10^6^)
Benin	2.323	0.988	42.5	1.161	1.440	1.488	1.516	2.323	2.323	2.183	2.125
Burkina Faso	4.099	1.969	48.0	2.049	2.916	2.978	3.006	4.099	4.032	4.032	4.024
Burundi	1.719	0.655	38.1	0.859	0.949	1.020	1.072	1.719	1.719	1.655	1.599
Cameroon	4.703	0.996	21.2	2.352	2.052	2.175	2.253	2.352	2.657	2.724	2.731
Côte d'Ivoire	4.621	1.775	38.4	2.311	2.835	2.790	2.908	4.621	4.254	4.179	3.870
Djibouti	0.041	0.009	21.3	0.020	0.020	0.020	0.020	0.020	0.029	0.028	0.025
Eritrea	0.818	0.341	41.7	0.409	0.639	0.547	0.563	0.818	0.808	0.746	0.727
Ethiopia	14.800	3.745	25.3	7.394	7.394	7.687	7.639	14.800	9.509	10.900	10.500
The Gambia	0.424	0.061	14.4	0.212	0.182	0.184	0.189	0.212	0.182	0.199	0.215
Ghana	5.395	2.945	54.6	5.395	4.676	4.618	4.470	5.395	5.395	5.395	5.333
Guinea	2.456	1.027	41.8	1.228	1.664	1.588	1.571	2.456	2.456	2.375	2.285
Guinea-Bissau	0.374	0.078	20.9	0.187	0.187	0.185	0.184	0.187	0.240	0.233	0.239
Kenya	6.881	2.682	39.0	3.441	3.989	4.484	4.492	6.881	6.533	6.126	5.827
Liberia	0.779	0.469	60.2	0.779	0.770	0.749	0.718	0.779	0.779	0.779	0.777
Malawi	2.692	1.276	47.4	1.346	1.980	1.725	1.862	2.692	2.692	2.692	2.665
Mali	3.577	1.758	49.1	1.789	2.814	2.680	2.654	3.577	3.566	3.488	3.415
Mauritania	0.792	0.218	27.5	0.396	0.396	0.403	0.405	0.792	0.601	0.604	0.589
Mozambique	4.264	2.495	58.5	4.264	3.748	3.686	3.660	4.264	4.264	4.260	4.236
Niger	3.944	0.851	21.6	1.972	1.835	1.844	1.863	1.972	2.561	2.540	2.472
Nigeria	35.300	15.000	42.5	17.700	22.800	23.100	23.000	35.300	31.500	32.200	32.000
Rwanda	1.872	0.627	33.5	0.936	0.936	1.067	1.064	1.872	1.584	1.636	1.575
Senegal	3.112	0.567	18.2	1.556	1.403	1.421	1.451	1.556	1.694	1.846	1.741
Sierra Leone	1.553	0.921	59.3	1.553	1.411	1.427	1.357	1.553	1.553	1.553	1.553
Somalia	1.770	0.685	38.7	0.885	1.006	1.030	1.019	1.770	1.657	1.690	1.649
Sudan	7.652	2.657	34.7	3.826	4.117	4.358	4.621	7.652	7.179	6.625	6.279
Tanzania	7.396	2.467	33.4	3.698	4.089	4.269	4.289	7.396	5.792	5.796	5.689
Togo	1.327	0.535	40.3	0.663	0.943	0.865	0.862	1.327	1.123	1.208	1.161
Uganda	6.315	1.309	20.7	3.158	3.158	3.113	3.160	3.158	3.866	3.942	3.700
Zambia	2.225	0.576	25.9	1.112	1.112	1.153	1.159	2.225	1.651	1.650	1.599
**TOTAL**	**133.223**	**49.680**	**37.3**	**72.650**	**81.463**	**82.654**	**83.027**	**123.765**	**112.195**	**113.282**	**110.601**

The estimates are based on the median schistosomiasis risk of the posterior predictive distribution for 29 West and East African countries. Treatment needs were calculated based on previously published geostatistical model-based prevalence estimates [2], [3] and WHO guidelines [4], [5].

### Annualized Praziquantel Treatment Needs

Table 1 summarizes the estimated amount of annualized praziquantel treatment needs, stratified by country, based on different administrative levels of risk estimates, i.e., country, province, district, and pixel level, using WHO thresholds. Table 2 shows the respective 50% CIs. Overall, in West and East Africa, 72.7 million treatments (50% CI: 68.8–100.7 million) are required if calculations are based on schistosomiasis risk estimates at country level. An additional 8.8 million treatments (resulting in a total of 81.5 million treatments, 50% CI: 67.3–107.5 million) are needed if the calculation is based on province-level risk estimates. Calculations employing risk estimates at higher level of disaggregation – at district or pixel level – result in a total amount of 82.7 million (50% CI: 66.0–111.1 million) and 83.0 million (50% CI: 61.0–119.8 million) treatments, respectively.

**Table 2:** Confidence intervals of praziquantel treatment needs for the school-aged population estimated at different spatial scales.

Country	Treatment needs (10% and 50% cut-offs)				Treatment needs (10% and 25% cut-offs)			
	Country-level (×10^6^)	Province-level (×10^6^)	District-level (×10^6^)	Pixel-level (×10^6^)	Country-level (×10^6^)	Province-level (×10^6^)	District-level (×10^6^)	Pixel-level (×10^6^)
Benin	1.161–2.323	1.161–2.172	1.184–2.215	1.166–2.170	2.323–2.323	1.877–2.323	1.612–2.323	1.404–2.323
Burkina Faso	2.049–4.099	2.188–4.032	2.158–4.078	2.079–4.071	4.099–4.099	3.827–4.099	3.733–4.099	2.865–4.099
Burundi	0.859–1.719	0.859–1.719	0.854–1.713	0.779–1.704	1.719–1.719	1.065–1.719	1.004–1.719	0.866–1.719
Cameroon	2.352–2.352	2.052–3.017	2.009–2.968	1.888–3.021	2.352–4.703	2.657–3.126	2.611–3.259	2.186–3.408
Côte d'Ivoire	2.311–2.311	2.311–3.982	2.302–4.127	2.146–4.326	4.621–4.621	3.267–4.621	3.205–4.621	2.411–4.569
Djibouti	0.020–0.041	0.018–0.029	0.017–0.031	0.015–0.033	0.020–0.041	0.018–0.041	0.017–0.040	0.015–0.040
Eritrea	0.409–0.818	0.409–0.818	0.409–0.818	0.409–0.818	0.409–0.818	0.409–0.818	0.409–0.818	0.409–0.818
Ethiopia	7.394–7.394	7.318–8.864	7.260–9.605	6.171–13.600	14.800–14.800	9.509–14.800	8.578–14.800	6.313–14.700
Gambia, The	0.212–0.212	0.161–0.212	0.160–0.212	0.157–0.216	0.212–0.212	0.161–0.324	0.160–0.310	0.157–0.338
Ghana	2.697–5.395	2.697–5.395	2.677–5.362	2.713–5.347	5.395–5.395	5.395–5.395	4.664–5.395	4.041–5.395
Guinea	1.228–2.456	1.228–2.269	1.252–2.375	1.170–2.379	2.456–2.456	1.964–2.456	1.715–2.456	1.397–2.454
Guinea-Bissau	0.187–0.187	0.160–0.212	0.159–0.232	0.142–0.246	0.187–0.374	0.160–0.374	0.159–0.368	0.143–0.365
Kenya	3.441–3.441	3.441–5.371	3.562–5.792	3.083–5.728	6.881–6.881	6.233–6.881	5.156–6.719	3.691–6.804
Liberia	0.389–0.779	0.435–0.779	0.420–0.779	0.403–0.778	0.779–0.779	0.779–0.779	0.711–0.779	0.599–0.779
Malawi	1.346–2.692	1.346–2.692	1.424–2.692	1.378–2.672	2.692–2.692	2.692–2.692	2.583–2.692	1.925–2.692
Mali	1.789–3.577	2.077–3.478	1.987–3.488	1.755–3.432	3.577–3.577	3.478–3.577	3.223–3.577	2.468–3.577
Mauritania	0.396–0.396	0.362–0.601	0.367–0.612	0.313–0.580	0.792–0.792	0.488–0.792	0.412–0.792	0.319–0.789
Mozambique	4.264–4.264	2.966–4.264	2.575–4.252	2.514–4.261	4.264–4.264	4.088–4.264	3.825–4.264	3.361–4.264
Niger	1.972–1.972	1.835–2.409	1.765–2.498	1.561–2.495	1.972–3.944	2.561–3.133	2.287–3.222	1.776–3.437
Nigeria	17.700–35.300	17.700–32.600	17.000–33.400	16.600–34.300	35.300–35.300	29.300–35.300	23.500–35.300	19.500–35.300
Rwanda	0.936–1.872	0.897–1.584	0.890–1.714	0.797–1.701	0.936–1.872	1.130–1.872	1.024–1.872	0.855–1.872
Senegal	1.556–1.556	1.403–1.507	1.326–1.537	1.255–1.772	1.556–1.556	1.694–2.125	1.707–2.234	1.430–2.216
Sierra Leone	0.776–1.553	0.776–1.553	0.776–1.553	0.783–1.553	1.553–1.553	1.411–1.553	1.427–1.553	1.170–1.553
Somalia	0.885–0.885	0.885–1.621	0.872–1.654	0.802–1.756	1.770–1.770	1.467–1.770	1.178–1.770	0.872–1.767
Sudan	3.826–3.826	3.826–5.217	3.997–6.065	3.222–7.468	7.652–7.652	7.179–7.652	5.881–7.652	3.296–7.646
Tanzania	3.698–3.698	3.879–5.301	3.880–5.506	3.321–6.114	7.396–7.396	5.251–7.388	4.979–7.237	3.791–7.258
Togo	0.663–1.327	0.663–1.123	0.663–1.110	0.626–1.137	1.327–1.327	1.123–1.327	0.943–1.327	0.731–1.298
Uganda	3.158–3.158	3.158–3.158	3.026–3.181	2.798–4.390	3.158–6.315	3.866–5.492	3.633–5.443	2.960–5.971
Zambia	1.112–1.112	1.112–1.474	1.055–1.519	0.906–1.778	2.225–2.225	1.651–2.225	1.248–2.190	0.926–2.196
**TOTAL**	**68.786–100.715**	**67.323–107.453**	**66.026–111.088**	**60.952–119.846**	**122.423–131.456**	**104.700–128.918**	**91.584–128.831**	**71.877–129.647**

The estimates are based on 50% confidence intervals of the posterior predictive distribution for 29 West and East African countries. Treatment needs were calculated based on previously published geostatistical model-based prevalence estimates [2], [3] and WHO guidelines [4], [5].

Over 5 million annual praziquantel treatments for the school-aged population are required in Ethiopia, Ghana, and Nigeria, while less than half a million treatments are needed for preventive chemotherapy in Djibouti, Eritrea, The Gambia, Guinea-Bissau, and Mauritania. These calculations are based on country-wide risk estimates and they vary when risk estimates are available at higher level of disaggregation.

Considering treatment in entire communities in high endemicity areas and 20% of the non-school-aged population in moderately endemic areas (as recently considered by WHO [17]), the required annual amount of praziquantel increases to more than 170 million or almost 235 million treatments using country- or pixel-level risk estimates, respectively (results not presented in Table 1).

### Country Examples

Next, selected country examples are given regarding annualized praziquantel treatment needs in the school-aged population. These examples highlight that the required amount of praziquantel depends on the level of aggregation of the schistosomiasis risk estimates, and is particularly pronounced in countries where the distribution of schistosomiasis shows high focality.

#### Ghana

**Figure 2:**
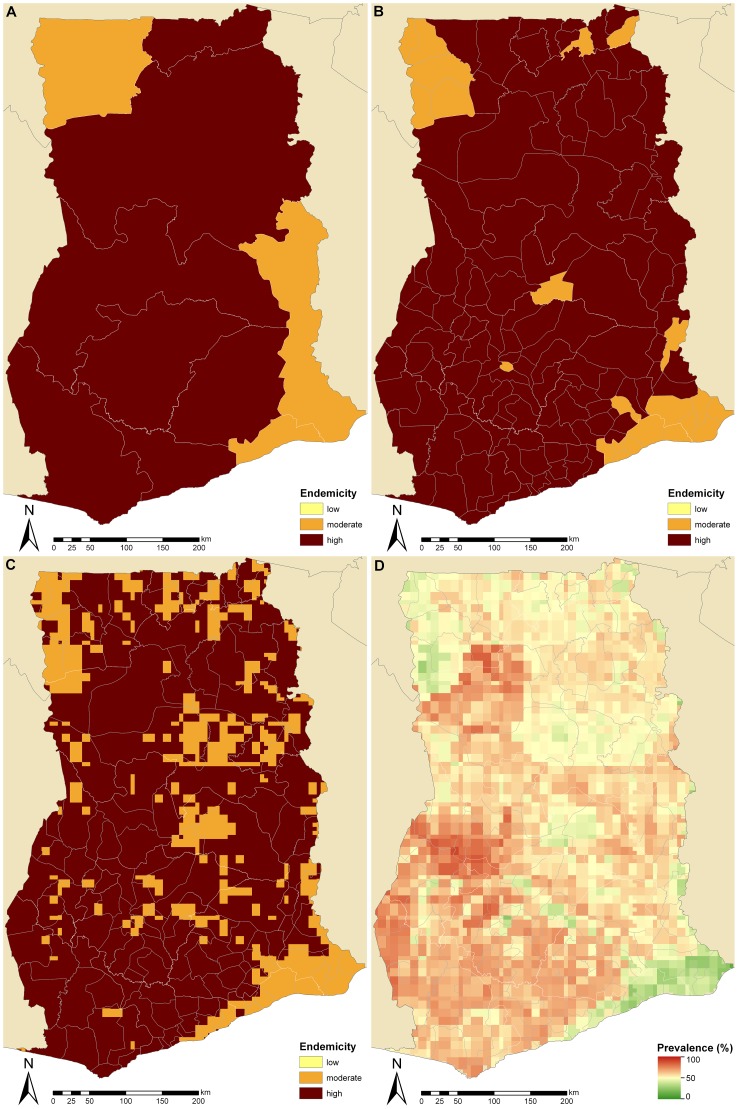
Schistosomiasis endemicity estimates at different administrative levels and pixel-level prevalence in Ghana. Provincial (A), district (B), and pixel-level (C) endemicity and pixel-level prevalence (D). The country-specific prevalence in Ghana is 54.6% based on previously published geostatistical model-based estimates [2], [3]. Low (schistosomiasis prevalence in school-aged children <10%), moderate (10–50%), and high (>50%) endemicity levels are based on WHO guidelines [4], [5].

Figure 2A displays endemicity classes based on population-adjusted prevalence of school-aged children at province level (called regions) throughout Ghana. The estimated prevalence in the Upper West and Volta regions is below the 50% threshold, even though Ghana was estimated to have an overall high endemicity of schistosomiasis with an average country-specific prevalence of 54.6%. Figures 2B and 2C show the endemicity classes at district and pixel level, respectively. Moderate levels of endemicity are found in some districts in the northeast, centre, and southeast of the country. A map of the spatial distribution of schistosomiasis infection risk at pixel level is given in Figure 2D. The lowest prevalence of 12.1% was detected in Ketu district of the Volta region (southeast Ghana) close to the border with Togo. The highest prevalence of 90.0% was found in the Sunyani and Wenchi districts of the Brong-Ahafo region (middle-western part of Ghana). Estimated amounts of annualized treatment needs for the school-aged population would be reduced from 5.4 million (50% CI: 2.7–5.4 million) to 4.7 million (50% CI: 2.7–5.4 million) when prevalence is aggregated at province- rather than country-level. The amount of treatments could be further reduced when aggregation is carried out at district level (4.6 million, 50% CI: 2.7–5.4 million) or pixel level (4.5 million, 50% CI: 2.7–5.4 million).

#### Burkina Faso

**Figure 3:**
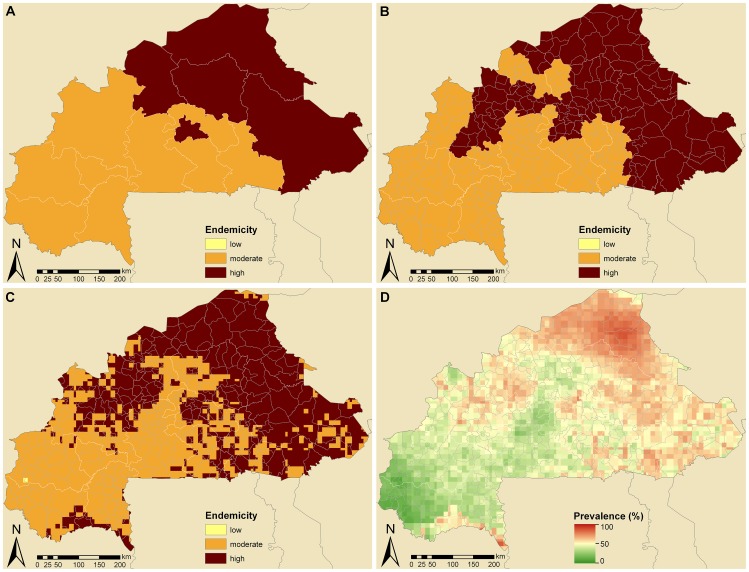
Schistosomiasis endemicity estimates at different administrative levels and pixel-level prevalence in Burkina Faso. Provincial (A), district (B), and pixel-level (C) endemicity and pixel-level prevalence (D). The country-specific prevalence in Burkina Faso is 48.0% based on previously published geostatistical model-based estimates [2], [3]. Low (schistosomiasis prevalence in school-aged children <10%), moderate (10–50%), and high (>50%) endemicity levels are based on WHO guidelines [4], [5].

Endemicity classes at province (called regions) and district level (called provinces) in Burkina Faso are shown in Figures 3A and 3B, respectively. The overall country-specific prevalence in school-aged children was 48.0%, indicating moderate endemicity. However, five regions in Burkina Faso (i.e., Centre, Centre-North, East, Nord, and Sahel) are highly endemic, as well as 20 (out of 45) communes. The endemicity-class map at pixel level (Figure 3C) further highlights that large parts of the country, especially in north-east and smaller areas in the south, are highly endemic, while a tiny part of the Hauts-Bassins region in Kénédougou province is classified as low-endemic. Figure 3D provides more details on the estimated spatial distribution of schistosomiasis risk throughout Burkina Faso, which varies from 9.0% to 87.5%. Annualized treatment needs would increase from about 2.0 million (50% CI: 2.0–4.1 million) to 2.9 million (50% CI: 2.2–4.0 million) with higher level aggregation of prevalence estimates (from country to province level) and remain at 3.0 million for both district and pixel level estimates (50% CI: 2.2–4.1 million and 2.1–4.1 million, respectively).

#### Zambia

**Figure 4:**
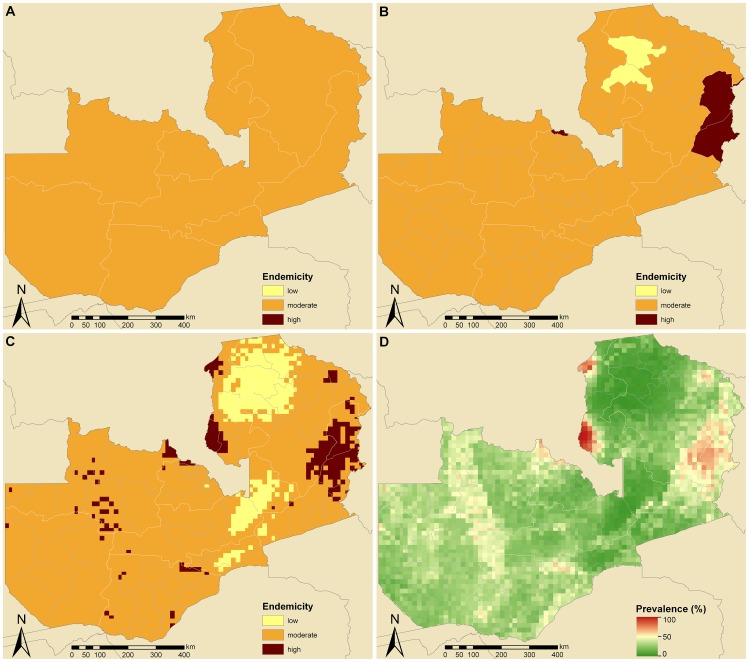
Schistosomiasis endemicity estimates at different administrative levels and pixel-level prevalence in Zambia. Provincial (A), district (B), and pixel-level (C) endemicity and pixel-level prevalence (D). The country-specific prevalence in Zambia is 25.9% based on previously published geostatistical model-based estimates [2], [3]. Low (schistosomiasis prevalence in school-aged children <10%), moderate (10–50%), and high (>50%) endemicity levels are based on WHO guidelines [4], [5].

The estimated country-specific prevalence of schistosomiasis in school-aged children in Zambia is 25.9%, which classifies the country as moderately endemic. A switch from country to province level revealed no changes in endemicity class (Figure 4A). However, at district-level (Figure 4B), Luwingo and Mporokoso (located in the north-east) are low-endemic, while the Chama, Chililabombwe, and Lundazi districts (in the east and centre-north) show high endemicity. Figures 4C and 4D show the distribution of endemicity classes and schistosomiasis risk in Zambia at pixel-level, respectively. The risk was estimated between 2.0% and 99.6%. Low-endemic areas are found in northern Zambia, covering about 250×250 km. Smaller low endemicity areas are located in the centre of the country. Highly endemic regions are distributed throughout Zambia with some larger clusters in the North and East. The estimated annualized treatment needs are between 1.1 and 1.2 million with only little effect of the aggregation level of schistosomiasis prevalence.

#### Senegal

**Figure 5:**
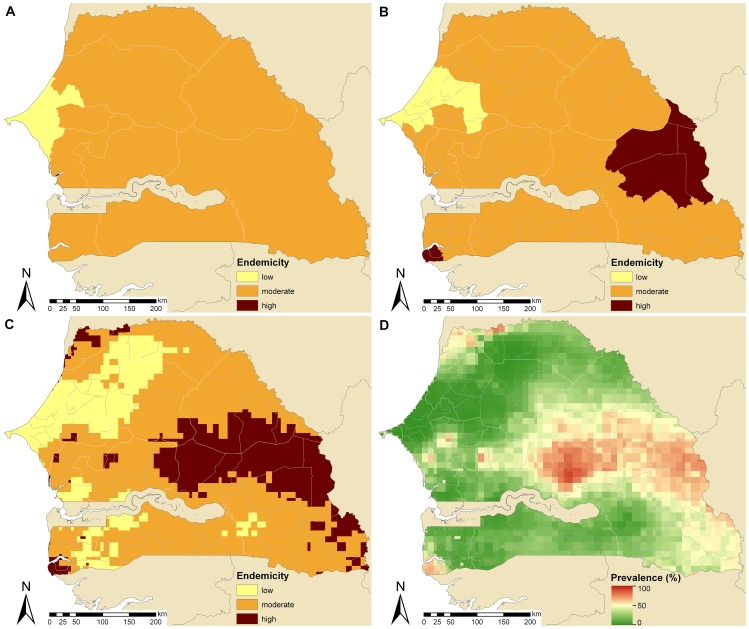
Schistosomiasis endemicity estimates at different administrative levels and pixel-level prevalence in Senegal. Provincial (A), district (B), and pixel-level (C) endemicity and pixel-level prevalence (D). The country-specific prevalence in Senegal is 18.2% based on previously published geostatistical model-based estimates [2], [3]. Low (schistosomiasis prevalence in school-aged children <10%), moderate (10–50%), and high (>50%) endemicity levels are based on WHO guidelines [4], [5].

Among the 29 countries included in the current analysis, Senegal has the second lowest national schistosomiasis prevalence in school-aged children (18.2%; moderately endemic). Figure 5A shows that the Dakar and Thiès regions (province level) have low endemicity, while the remaining regions are moderately endemic. District level (Figure 5B) and pixel level (Figure 5C) maps indicate that areas of low endemicity expand from the West of Senegal toward the North. Some areas are classified as highly endemic, mainly in the East. The spatial distribution of schistosomiasis risk over Senegal varies from 0.5% (region of Thiès) to 89.3% (Tambacounda department in the Tambacounda region, central Senegal) as shown in Figure 5D. About 1.6 million praziquantel treatments (50% CI: 1.6–1.6 million) are required yearly according to population-adjusted schistosomiasis prevalence estimated at country-level. An estimated 1.4 million treatments are needed based on province- or district-based data aggregation (50% CI: 1.4–1.5 million and 1.3–1.5 million, respectively). According to pixel-based approaches, there are an estimated 1.5 million treatments (50% CI: 1.3–1.8 million) needed every year.

### Effect of Changing Thresholds

There are ongoing discussions within WHO and other organizations and consortia whether the threshold to separate between moderate and heavy schistosomiasis endemicity should be halved from 50% to 25%. Of note, the lower threshold is already adopted by the Schistosomiasis Consortium for Operational Research and Evaluation (SCORE) in the frame of large-scale programs that aim at gaining (prevalence ≥25%) and sustaining (prevalence 10–24%) schistosomiasis control (http://score.uga.edu/Projects.html). This new 25% threshold would imply that more than 120 million annualized praziquantel treatments (50% CI: 122.4–131.5 million) would be needed in the 29 African countries for the school-aged population alone when considering country-level prevalence estimates, and around 112 million treatments based on aggregations at other administrative levels (see Tables 1 and 2). A change in the threshold would not affect all countries in the same way, e.g., the estimated total praziquantel treatment needs in The Gambia (with an averaged population-adjusted prevalence of 14.4%), Ghana (54.6%), Mozambique (58.5%), Sierra Leone (59.3%), and Liberia (60.2%) would remain almost the same, irrespective of the level of aggregation, while it would increases by more than 50% in Rwanda (33.5%), Sudan (34.7%), Burundi (38.1%), Somalia (38.7%), and Malawi (47.4%) based on prevalence aggregations at district-level.

## Discussion

Preventive chemotherapy is the current mainstay for morbidity control due to schistosomiasis and other helmintic diseases [4], [9], [14], [18]–[20]. The administration of praziquantel to high-risk communities, most importantly school-aged children, is often based on crude *Schistosoma* prevalence estimates that are aggregated at an arbitrarily chosen administrative level. Our analysis shows that the geographical scale at which schistosomiasis prevalence data are aggregated affects the estimated amount of treatment needs, and hence has to be considered in the calculation of the number of praziquantel treatments required for national schistosomiasis control programs.

Contrary to what we expected, the total amount of praziquantel treatments is higher at a smaller administrative unit of schistosomiasis prevalence aggregation (i.e., district level *versus* province or country level). For example, we estimate that the annualized praziquantel treatment needs for the school-aged population are more than 10% higher in the ensemble of 29 West and East African countries considered here when calculated using district instead of country level prevalence estimates. This observation can be explained because many East and West African countries had a prevalence of schistosomiasis in school-aged children at around 40% (moderately endemic situation), and the infection risk is not uniformly distributed throughout a country. Indeed, some province or district specific prevalence estimates were above the 50% threshold suggested by WHO, and hence warrant annual treatment rather than treatment every second year for moderate endemicity areas (prevalence 10–50%). We rarely observed switches from moderate to low endemicity levels. Such switches are expected to occur in countries where the overall prevalence of *Schistosoma* infection is close to 10%. Such low prevalence levels were only found in The Gambia (14.4%) and Senegal (18.2%). In countries currently classified as highly endemic, the required amount of treatment per year will be reduced if schistosomiasis prevalence is calculated at province or even district level, as some areas will fall under the 50% threshold.

Utzinger et al. (2009) [10] estimated that 91.3 million praziquantel treatments are needed for the school-aged population based on risk estimates aggregated at country level with 2006 population data for the 29 East and West African countries considered here. This is almost 20 million more than the 72.7 million treatments estimated in the current study. What are the reasons for this difference? Most importantly, there are marked differences in the country prevalence estimates of schistosomiasis leading to different classifications of endemicity (e.g., Burkina Faso, Mali, and Tanzania). Utzinger and colleagues employed prevalence estimates published in Steinmann et al. (2006) [1], which are to a large extent based on data obtained before 1989. However, due to major demographic and ecological transformations (e.g., urbanization), social and economic development, and implementation of schistosomiasis control programs, country prevalence estimates have changed (mostly to lower levels). Moreover, different sources of population data and the estimated percentage of school-aged children have some leverage on the estimated annualized praziquantel treatment needs.

In early 2012, WHO released new estimates on the total number of individuals requiring preventive chemotherapy with praziquantel [17]. For Africa (using WHO country classifications; e.g. Sudan being part of the Eastern Mediterranean rather than Africa), it is estimated that as many as 102 million treatments are needed for the school-aged population and 221 million treatments when including communities in high and moderately endemic areas. Of note, secondary administrative level schistosomiasis risk estimates were employed to define endemicity levels. The bulk of these risk estimates lacks geostatistical modeling and neglects potential spatial correlation inherent to the data. Moreover, in the absence of recent data, information was also obtained from surveys conducted before 1989 without accounting for changes in prevalence levels over time. Compared to the WHO treatment needs calculations for the school-aged population, our estimates are by approximately 30 million annual treatments smaller, but we used a different set of African countries in the current analysis. For the missing African countries we used the previously published country-level prevalence estimates [10] to calculate the total treatment needs in the school-aged population. This assumption would result in an annualized amount of 105.9 million praziquantel treatments per year in school-aged children. Interestingly, this estimate is very close to the 102 million put forth by WHO [17].

Several issues warrant discussion regarding our geostatistical model-based analysis and its implication for preventive chemotherapy. First, human, financial, and technical resources for the control of neglected tropical diseases, including schistosomiasis, have increased considerably in recent years [21], [22]. New initiatives and partnerships aim to bring praziquantel distribution to scale, such as the Schistosomiasis Control Initiative (SCI) [19] and the Global Network for Neglected Tropical Diseases (http://www.globalnetwork.org/) [10]. It is therefore conceivable that the overall schistosomiasis prevalence in some areas might have dropped from high to moderate, and from moderate to low endemicity thresholds, which would result in fewer treatments required. However, rapid re-infection after de-worming is a common phenomenon, which challenges the control of schistosomiasis [23] and other helminth infections [24], [25], and hence has to be considered when reducing the frequency of treatment campaigns. Some experts are currently discussing to lower the threshold distinguishing between moderate and high endemicity from 50% to 25% to further reduce morbidity and this strategy might also have an impact on transmission. However, such a move would strongly increase the required amount of annualized treatment needs for the school-aged population from 72.7 million to more than 120 million for the 29 West and East African countries considered here.

Our praziquantel treatment needs were calculated based on schistosomiasis risk estimates summarized at different scales. Administrative boundaries were used because large-scale preventive chemotherapy programs are usually planned and conducted at specific administrative levels (e.g., district). However, given the strong focality of schistosomiasis, there is strong heterogeneity within administrative boundaries. For instance, villages in close proximity to freshwater bodies, where intermediate host snails proliferate, are often highly endemic for schistosomiasis, while villages farther away (but in the same administrative region) might have prevalence levels below 10%, even though the overall endemicity in the considered administrative unit is moderate. Hence, stratification of areas should preferably be based on the geography and suitable freshwater bodies (e.g., ecozones), which is the recommended strategy by WHO [4]. However, many schistosomiasis data are not (yet) available at geographically stratified locations, but are instead aggregated over administrative levels. This makes it difficult for local health managers to implement preventive chemotherapy programs considering the environment. In addition, stratification of the area according to the environment is more complex since multiple ecological factors may interact.

Large parts of Africa are co-endemic for several neglected tropical diseases [26]–[29]. This is explained by similar pathways of infection and lack of preventive measures [30], [31]. Hence, efforts should be made to integrate control programs so that multiple neglected tropical diseases can be addressed in order to reduce co-morbidity and costs. The morbidity of up to seven neglected tropical diseases might be addressed by preventive chemotherapy with four drugs, which can be safely co-administered based on the current state of knowledge [18], [32], [33]. However, with decreasing level of co-infection risk due to reduced prevalence, co-administration of multiple drugs becomes less cost-effective and many individuals will be treated unnecessarily with multiple drugs [34], [35]. This potentially increases the risk of drug resistance development.

Considering that, on average, three tablets of praziquantel are needed to treat a school-aged child for schistosomiasis, more than 210 million tablets would need to be administered every year to this population group in the 29 East and West African countries considered here. The distribution of such large amounts of praziquantel is a formidable challenge, especially in remote rural areas. It will require strong health systems and good supply strategies, which are currently lacking in large parts of Africa. Even though the price of a single tablet of praziquantel is now below US$ 0.10 [36], [37] and pharmaceutical companies have pledged new donations, the costs for drug storage and distribution are considerable. Additionally, it might be hard to achieve high compliance after several rounds of preventive chemotherapy, because morbidity is likely to be reduced and individuals might refuse to take the drugs when feeling healthy.

With the transition from morbidity control to transmission control of schistosomiasis and other helminthic diseases, preventive chemotherapy alone is unlikely to achieve this goal [38], [39]. Complementary measures will be required depending on the setting and available resources. For instance, the “Tokomeza Kichocho” project is aiming to eliminate schistosomiasis from Zanzibar based on a multi-pronged approach, i.e., preventive chemotherapy targeting school-aged children, intermediate host snail control, improved access to sanitation, water and hygiene, and formative health education to change human behavior.

In conclusion, the geographical scale to estimate schistosomiasis risk should take into consideration the expected country-specific prevalence level and the diversity of schistosomiasis risk in the country in order to accurately calculate praziquantel treatment needs. In countries with rather homogeneous schistosomiasis distribution, it would be sufficient to implement control strategies based on schistosomiasis prevalence aggregated at country level. However, in countries with a highly focal distribution of schistosomiasis, province- or even district-level calculations of prevalence have to be considered in order to provide sufficient amounts of treatments to those at risk of morbidity and to reduce the costs in areas requiring less frequent rounds of preventive chemotherapy. The work reported here can be readily adapted to other neglected tropical diseases that have also endorsed preventive chemotherapy as the strategy of choice for morbidity control. In addition, it will be interesting to repeat our analysis in the frame of ongoing large-scale control programs, which are constantly changing prevalence levels and disease, and hence treatment needs.
